# Posttraumatic stress disorder symptoms among child survivors of the 2008 Wenchuan earthquake: a comparison between Chinese ethnic Han and Hui groups

**DOI:** 10.7717/peerj.11967

**Published:** 2021-08-11

**Authors:** Xiacan Chen, Bin Li, Wan-Jun Guo, Jia-Jun Xu

**Affiliations:** 1 Institute of Forensic Medicine, West China School of Basic Medical Sciences and Forensic Medicine, Sichuan University, Chengdu, China; 2 Mental Health Center, West China Hospital Sichuan University, Chengdu, China

**Keywords:** Posttraumatic stress disorder, Child, Adolescent, Ethnicity, Earthquake

## Abstract

**Background:**

Relatively few studies have compared posttraumatic stress disorder (PTSD) symptoms following a disaster among children of different ethnicities. We sought to investigate the differences in PTSD symptoms between the ethnic Hui and Han child survivors of the 2008 Wenchuan earthquake in China.

**Methods:**

This study collected data from 1,951 Han and 247 Hui child survivors of the 2008 Wenchuan earthquake in China. The children ranged from 7 to 15 years of age. Earthquake-related exposures were measured using a modified version of the PsySTART Rapid Triage System. PTSD symptoms were evaluated using the University of California, Los Angeles PTSD-Reaction Index (UCLA PTSD-RI). Personality characteristics were assessed using the Junior Eysenck Personality Questionnaire (JEPQ). Multiple linear regression was used to investigate the association between the ethnicity and the severity of PTSD symptoms. Multiple logistic regression was used to investigate the association between the ethnicity and the percentage of screening positive for PTSD symptoms.

**Results:**

The average UCLA PTSD-RI total score of the ethnic Hui group (27.01 ± 9.24) was significantly higher than that of the ethnic Han group (25.12 ± 9.17) (t = −3.05, *p* = 0.002), as were the avoidance/numbness (Hui: 10.02 ± 4.82; Han: 9.04 ± 4.60, t = −3.12, *p* = 0.002) and arousal scores (Hui: 9.36 ± 3.64; Han: 8.79 ± 3.42, t = −2.44, *p* = 0.015). The percentage of screening positive for D criteria (arousal symptoms) also differed significantly between the ethnic Han (41.9%, 95% CI [39.7–44.1%]) and Hui (48.6%, 95% CI [42.3–54.9%]) groups (χ^2^ = 3.97, *p* = 0.046). Ethnicity was associated with the avoidance/numbness symptom score following adjustments for sex, age, personality traits and earthquake exposure experiences by multiple linear regression (B: 0.61, 95% CI [0.04–1.17], *p* = 0.035). The initial significant associations between the ethnicity and the arousal symptoms score and the PTSD total score disappeared while adjusting for the subjective earthquake exposure experiences (Model 5: arousal symptoms, B = 0.41, 95% CI [−0.01 to 0.83], *p* = 0.056; PTSD, B = 1.00, 95% CI [−0.07 to 2.07], *p* = 0.066). The initial significant association between the ethnicity and the percentage of screening positive for D criteria disappeared while adjusting for the objective earthquake exposure experiences (Model 4: OR = 1.32, 95% CI [1.00–1.75], *p* = 0.052).

**Conclusion:**

This study is the first to report the relationship between the ethnicity and PTSD symptoms among child survivors following a disaster. The findings of this study suggest that the trauma-focused cognitive behavior therapy could also be an effective treatment for Chinese ethnic Hui and Han children who are suffering from PTSD. Future research could be designed to examine whether cultural differences in perceptions and interpretations may account for the variations in subjective experiences. More attention should be paid to the ethnic minority children with PTSD in the future.

## Introduction

Posttraumatic stress disorder (PTSD) is a common mental disorder that occurs following the exposure to one or more traumatic events, especially a disaster that poses a significant burden to individuals (Campbell et al., 2007). The essential feature of PTSD is the development of characteristic symptoms, including intrusion, avoidance, negative cognition/mood and heightened arousal. These symptoms may last from several weeks up to years (Neria, Nandi & Galea, 2008), especially for children who are highly vulnerable to develop PTSD symptoms after disasters (Gurwitch et al., 2004). Research has shown that the level of severity of PTSD symptoms has been associated with ethnicity in children (Andrews et al., 2015; Das-Munshi et al., 2016; Perilla, Norris & Lavizzo, 2002; Roberts et al., 2011). Research on distinct PTSD symptoms among ethnic groups has been conducted on White, African-American, Hispanics and non-Hispanic adults (Perilla, Norris & Lavizzo, 2002; Roberts et al., 2011), as well as among ethnic Qiang and Han adults in China (Chen et al., 2011; Kun et al., 2013). However, the differences in PTSD symptoms among Chinese children of distinct ethnicities have not been studied.

The Hui is a minority ethnic group in China (NBSC, 2010) whose main religion is Islam. The Han is the majority ethnic group in China, influenced by different cultures and sources, and has formed a variety of religious beliefs that have developed over thousands of years (Cao & Wang, 2020; Xu, 2010). Some studies have shown that the differences in PTSD symptoms are related to culturally of distinct ways of thinking and behaving that affect the psychological landscape of the self through a strengthening of specific neural processes and consolidation of particular cognitive, emotional and behavioral patterns (Han & Northoff, 2008; Han et al., 2013; Kitayama & Uskul, 2011). Therefore, we hypothesize that the ethnic Hui and Han children may show different PTSD symptoms. Culturally-sensitive treatment of PTSD could be recommended to accommodate the possible variations among ethnic groups (Roberts et al., 2011). The various manifestations and severity of PTSD symptoms demand the use of tailored psychological intervention strategies, according to the American Academy of Child and Adolescent Psychiatry (Cohen, 2010). Consequently, we wish to determine whether PTSD symptoms differ between the ethnic Hui and Han children in order to provide information relevant to the treatment for ethnic Hui children.

Although there have been many reports on PTSD among immigrants and ethnic minorities (Perilla, Norris & Lavizzo, 2002; Roberts et al., 2011; Chen et al., 2011; Kun et al., 2013), different traumatic events may cause different levels of traumatic stress. Therefore, it is challenging to compare stress responses between ethnic groups. The 2008 Wenchuan earthquake in Sichuan province, China is a devastating earthquake measured 8.0 on the Richter scale (Stone, 2008). It has caused 69,195 deaths until July 1, 2008 (UNICEF, 2008). Qingchuan County was one of the areas being most severely affected by the 2008 Wenchuan earthquake. There are two Hui Autonomous Townships in the Qingchuan County, which are populated by Hui and Han people who have lived together (Zhou, 1996). The shared macro-level environmental, spatial and socioeconomic conditions between ethnic Hui and Han groups living there make for a unique opportunity to compare PTSD symptoms between ethnic groups.

Disaster exposure experiences—both objective and subjective—are correlated with the PTSD symptoms in children (Perrin, Smith & Yule, 2000; Trickey et al., 2012). Objective disaster exposure experiences of children were reported as weak or moderate risk factors for PTSD symptoms (Roussos et al., 2005), whereas subjective disaster exposure experiences of children were reported as strong risk factors for PTSD symptoms (Chen et al., 2017; Thienkrua et al., 2006; Trickey et al., 2012). The distinct characteristics of Hui and Han groups may correspond to a difference in their subjective earthquake exposure experiences, even if the two groups of children came from the same stricken area. However, the subjective earthquake exposure experiences have not been reported for ethnic Hui and Han children. Moreover, PTSD symptoms following a disaster have been associated with demographic and environmental factors, personality traits and disaster exposure experiences; all of these factors could influence the relationship between the ethnicity and PTSD symptoms (Ekşi et al., 2007; Thienkrua et al., 2006; Ying et al., 2013). Therefore, it is imperative to consider the effect of these factors during an evaluation of the relationship between the ethnicity and PTSD symptoms.

Considering these considerations, we sought to determine whether or not the PTSD symptoms differed between the Hui minority and the Han majority child survivors, and to explore the relationship between ethnicity and PTSD symptoms while adjusting for other factors. We anticipate that the findings from this study may provide information that may assist the development of appropriate psychological intervention strategies designed specifically for ethnic Hui children with PTSD.

## Materials & methods

### Procedures

This present research is a secondary analysis that draws upon a sub-sample from a large cross-sectional survey conducted in 2009 (one year after the Wenchuan earthquake). The survey included 21,652 children between 7 and 15 years old who were survivors of the Wenchuan earthquake and who lived in Qingchuan County, Guangyuan City, at the time of the survey. Qingchuan County was one of areas being most severely affected by the 2008 Wenchuan earthquake. The survey was commissioned by the local department of education to screen for mental health problems in order to provide the appropriate mental health services. The survey was administered in the school through face-to-face interviews by trained interviewers who were psychiatrists, psychologists, psychiatric nurses, or social workers. A detailed description of the methods and recruitment for the survey have been described in a previous work (Chen et al., 2017; Xu et al., 2012). Written informed consent was provided by the parents or legal guardians of the children during the data collection and the survey was performed in accordance with the Declaration of Helsinki. The ethics approval for the secondary analysis of the data for research purposes was obtained from the Medical Ethics Committee of West China Hospital (No. 2015-52).

### Samples

**Figure 1:**
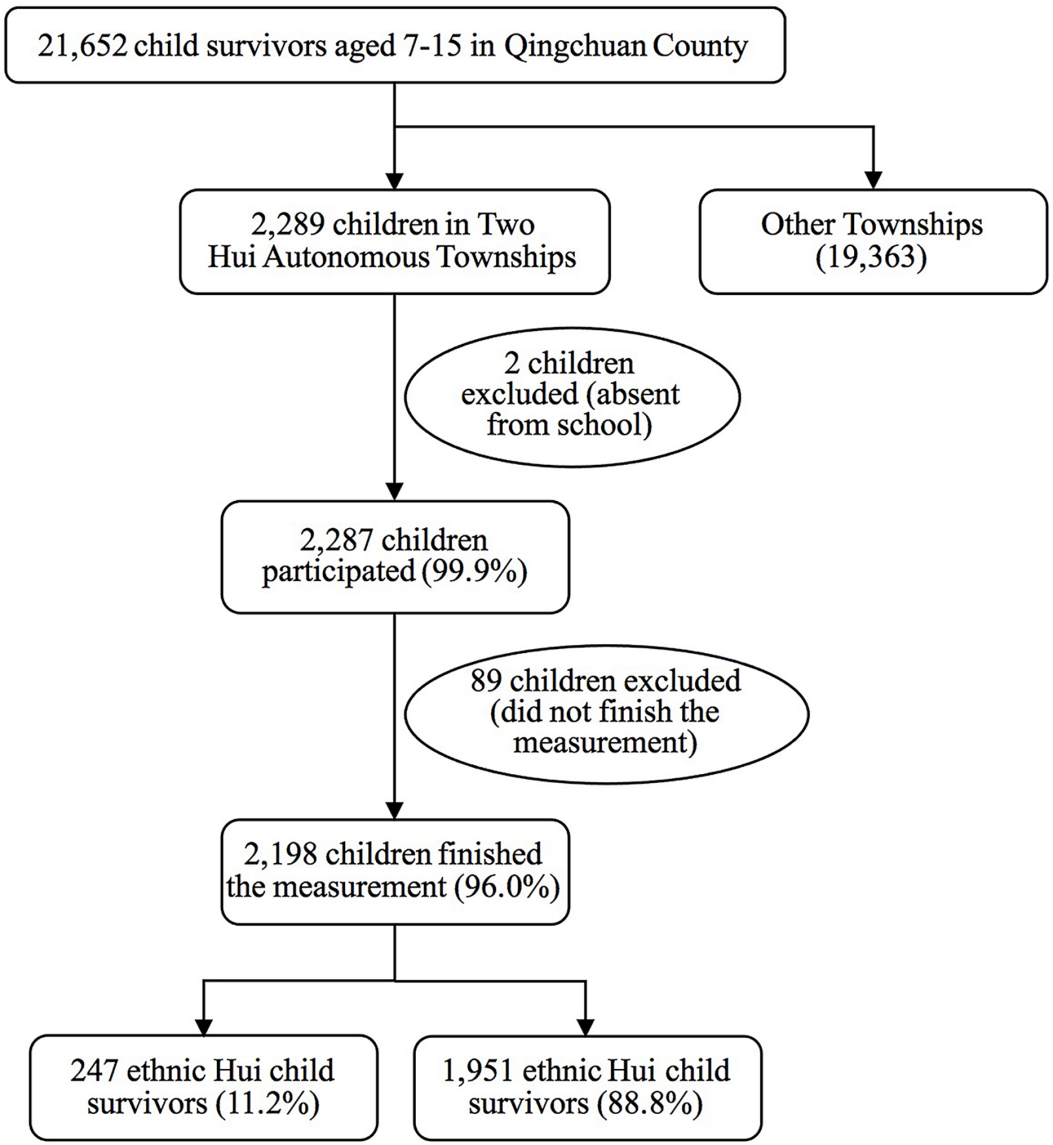
Sample selection process for child survivors in Hui Autonomous Townships, Qingchuan County.

In this secondary analysis study, we examined the data on children from two Hui Autonomous Townships of Qingchuan County. The sample selection process is illustrated in Fig. 1. The Hui Autonomous Townships are populated by Hui and Han people who have lived together (Zhou, 1996) under similar socioeconomic conditions. The two groups share similar macro-level environmental, spatial and socioeconomic conditions of the two ethnic groups. The children of the Hui and Han ethnic groups accounted for 99.9% (*n* = 2,287) of all of the children (*n* = 2,289) in the two autonomous townships. A total of 2,198 children (96.0%) completed the interview of survey. The Han people represent the largest ethnic group and the Hui are one of the main ethnic minorities in China. Due to their numbers in the affected regions, Hui people were the ethnic minority most heavily impacted by the Wenchuan earthquake in the autonomous townships. The survey included 426 Hui children aged between 7 to 15 who were living in Qingchuan County. A total of 247 lived in two Hui Autonomous Townships and the others (*n* = 179) resided in various parts of Qingchuan County. The total sample of this study consisted of 247 (11.2%) ethnic Hui children and 1951 (88.8%) ethnic Han children in the two autonomous townships. Data on the participants’ sex, age, place of residence, and resettlement following the earthquake are presented in Table 1.

**Table 1:** Comparison of demographic characteristics and personality traits between the ethnic Hui and Han of children groups following the Wenchuan earthquake.

Variables	Total *N* = 2,198 (%)	Han *n* = 1,951 (%)	Hui *n* = 247 (%)	χ^2^/Mann–Whitey U	*P*
Male, *n* (%)	1,075 (48.91)	933 (47.82)	142 (57.49)	8.20	0.004
Age group, *n* (%)					
7–11 years	952 (43.31)	831 (42.59)	121 (48.99)	3.65	0.056
12–15 years	1,246 (56.69)	1,120 (57.41)	126 (51.01)		
Rural residents, *n* (%)	2,019 (91.86)	1,795 (92.00)	224 (90.69)	0.51	0.476
Resettled after earthquake, *n* (%)	907 (41.26)	780 (39.98)	127 (51.42)	11.83	0.001
P score, median (IQR)	49.79 (14.70)	49.75 (15.06)	50.39 (14.23)		0.472
Psychoticism, *n* (%)	158 (7.19)	134 (6.87)	24 (9.72)	3.41	0.492
Moderate psychoticism, *n* (%)	302 (13.74)	268 (13.74)	34 (13.76)		
Intermediate, *n* (%)	1,081 (49.18)	959 (49.15)	122 (49.39)		
Moderate socialization, *n* (%)	237 (10.78)	211 (10.81)	26 (10.53)		
Socialization, *n* (%)	420 (19.11)	379 (19.43)	41 (16.6)		
E score, median (IQR)	42.63 (14.03)	42.58 (14.45)	43.41 (13.25)		0.353
Extraversion, *n* (%)	737 (33.53)	660 (33.83)	77 (31.17)	5.43	0.246
Moderate extraversion, *n* (%)	413 (18.79)	367 (18.81)	46 (18.62)		
Intermediate, *n* (%)	901 (40.99)	792 (40.59)	109 (44.13)		
Moderate introversion, *n* (%)	102 (4.64)	88 (4.51)	5.67 (14)		
Introversion, *n* (%)	45 (2.05)	44 (2.26)	0.4 (1)		
*N* score, median (IQR)	51.75 (14.72)	51.70 (14.86)	52.26 (14.77)		0.735
Neuroticism, *n* (%)	8.42 (185)	167 (8.56)	7.29 (18)	3.64	0.457
Moderate neuroticism, *n* (%)	13.33 (293)	267 (14.00)	26 (11.00)		
Intermediate, *n* (%)	45.31 (996)	878 (45.00)	118 (47.77)		
Moderate stability, *n* (%)	14.24 (313)	272 (13.94)	41 (16.60)		
Stability, *n* (%)	18.70 (411)	367 (18.81)	44 (17.81)		
L score, median (IQR)	48.11 (14.88)	47.52 (15.17)	49.29 (14.26)		0.093
Innocence, *n* (%)	430 (19.56)	387 (19.84)	43 (17.41)	2.70	0.610
Moderate innocence, *n* (%)	290 (13.19)	263 (13.48)	27 (10.93)		
Intermediate, *n* (%)	1010 (45.95)	891 (45.67)	119 (48.18)		
Moderate deception, *n* (%)	302 (13.74)	264 (13.53)	38 (15.38)		
Deception, *n* (%)	166 (7.55)	146 (7.48)	20 (8.10)		

**Note:**

Abbreviations: Han, the Han ethnic child group; Hui, the Hui ethnic child group; IQR, interquartile range; *P*, psychoticism/socialization subscale; E, extraversion/introversion subscale; *N*, neuroticism/stability subscale; L, lie subscale.

### Measurements

The demographic variables, including sex, age, place of residence (rural or urban), and resettlement following the earthquake, were collected by a self-administered questionnaire.

An earthquake-modified version of the PsySTART Rapid Triage System was used to assess the earthquake exposure experiences of children (Gurwitch et al., 2004; Pynoos, Schreiber & Steinberg, 2004; Thienkrua et al., 2006). This system included 11 yes/no questions adapted from an earthquake exposure scale informed by the Diagnostic and Statistical Manual of Mental Disorders IV (DSM-IV) A-1 and A-2 criteria for PTSD. The system is composed of 6 items that serve to identify objective features of earthquake exposure (*i.e*. having experienced an earthquake, having been injured, having seen people die or being injured, having lost close family member or friend, having close family members or friends injured, having lost one’s home or important belongings), and 5 items that serve to identify subjective exposures (*i.e*. having felt unable to escape from the disaster, having felt panic of others, having felt extreme panic or fear, having been trapped for a longer time, having felt one’s own or a family member’s life to have been in danger) (Chen et al., 2017). Participants were instructed to answer “yes” or “no” to each of the items. All the children had experienced the Wenchuan earthquake, and all of the participants answered “yes” to the item of “having experienced an earthquake” in this study. Therefore, this item is not listed in Table 1 and was not considered in the subsequent analysis.

The adolescent version of the University of California, Los Angeles PTSD-Reaction Index (UCLA PTSD-RI) has been widely used to assess traumatized children following major disasters and catastrophic violence (Steinberg et al., 2004). The index has been translated into Chinese and was used to evaluate the symptoms of PTSD in our study. It contains 20 items that assess intrusion, avoidance/numbness, and arousal symptoms. Each item is rated on a 5-point scale ranging from “never” (0) to “most of the time” (4). The Cronbach’s α was 0.91, 0.79, 0.80 and 0.74 for the total scale, intrusion symptoms, avoidance/numbness symptoms and arousal symptoms, respectively (Chen, Hung & Lin, 2000; Chen et al., 2002; Zhang, 2006). If the frequency of any intrusion symptoms assessment item had a value of greater than or equal to 3, the intrusion symptom (B criteria) was positive (percentage of screening positive for B criteria). If the frequency of any three avoidance/numbness symptoms assessment items was greater than or equal to 3, the avoidance/numbness symptom (C criteria) was positive (percentage of screening positive for C criteria). If the frequency of any two arousal symptoms assessment item was greater than or equal to 3, the arousal symptom (D criteria) was positive (percentage of screening positive for D criteria). If the symptoms of a participant met all the B, C and D criteria, the subject met the diagnostic criteria of DSM-IV and was screened positive for PTSD in this study (Zhang, 2006).

The Junior Eysenck Personality Questionnaire (JEPQ) is a self-report questionnaire for children aged 7 to 15 (Claridge, 1977; Eysenck, Barrett & Saklofske, 2020). Because it was translated into Chinese and modified in the 1980s (Chen, 1983; Gong, 1984a), it has been widely used in China over the past 30 years (Gong, 1984b; Zhu et al., 1983). The Chinese version of the JEPQ, which was used to assess personality characteristics in our study, was comprised of 88 yes/no items (scored as “yes” = 1, “no” = 0) with four sub-scales: psychoticism/socialization (P), extraversion/introversion (E), neuroticism/stability (N) and lie (L). The Cronbach’s α coefficient of the P, E, N and L subscales and the JEPQ were 0.80, 0.83, 0.62 and 0.78, respectively (Gong, 1984b; Lyu, 2008). The score of each subscale was divided into five levels. For instance, the P subscale was divided into socialization (T < 38.5), moderate socialization (38.5 ≤ T < 43.3), intermediate (43.3 ≤ T ≤ 56.7), moderate psychoticism (56.7 < T ≤ 61.5) and psychoticism (T > 61.5).

### Data analysis

A normal Q–Q plot was used to observe the normal distribution of the continuous variables. The t-test was used to compare the continuous variables with a normal distribution. The Mann–Whitney U test was used to compare the continuous variables that did not conform to a normal distribution. The chi-square test was used to examine the difference of categorical variables between the two ethnic groups. The PTSD symptoms which had statistical significance between the two ethnic groups were included as dependent variables in the subsequent regression analyses. The multiple linear regression was used to investigate the association between the ethnicity (independent variable) and the severity of PTSD symptoms based on the UCLA PTSD-RI score (dependent variable). Multiple logistic regression analysis was used to investigate the association between ethnicity (independent variable) and the percentage of screening positive for PTSD symptoms (dependent variable). The demographic variables, the JEPQ scores of the four personality traits, the objective earthquake exposure experiences and the subjective earthquake exposure experiences were included as independent variables step by step. The regression analysis yielded B or odds ratio with a 95% confidence interval (CI) for each independent variable. All tests were two-tailed and an alpha level of below 0.05 was considered to be statistically significant. All statistical analyses were performed using the SPSS 16.0.

## Results

### Demographic

Sex and resettlement to another area after the earthquake were variables that differed significantly between the Hui and Han ethnic child groups (Table 1). Age differed significantly between the Hui (median: 12.0, interquartile range: 4.0) and Han (median: 12.0, interquartile range: 4.0) ethnic child groups (U = 216,585.50, *p* = 0.009), while age groups did not (Table 1).

### Personality characteristics

The median (interquartile range, IQR) of the P (U = 234,192.00, *p* = 0.472), E (U = 232,213.50, *p* = 0.353), N (U = 237,763.00, *p* = 0.735), and L (U = 225,181.50, *p* = 0.093) subscales did not differ significantly between the ethnic Hui and Han child groups. Each subscale was divided into five levels and the distribution of the levels did not differ significantly between the ethnic Hui and Han child groups in each subscale of the JEPQ (Table 1).

### Earthquake exposure experiences

**Figure 2:**
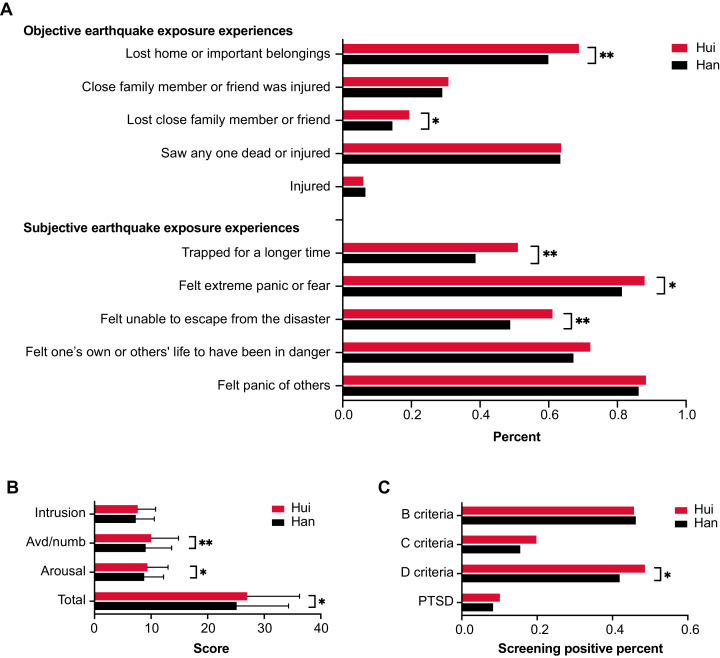
Comparison of earthquake exposure experiences (A), UCLA PTSD-RI scores (B) and percentage of screening positive for PTSD symptoms (C) for the ethnic Hui and Han groups of children. Abbreviations: *, *p* < 0.01; **, *p* < 0.05; Avd/numb, avoidance/numbness; PTSD, posttraumatic stress disorder; Han, the Han ethnic child group; Hui, the Hui ethnic child group.

Three subjective earthquake exposure experiences showed statistically significant differences between the Hui and Han ethnic child groups (Fig. 2): having been trapped for a longer time (Han: 755, 38.7%; Hui: 126, 51.0%), having felt unable to escape from the disaster (Han: 952, 48.8%; Hui: 151, 61.1%), and having felt extreme panic or fear (Han: 1587, 81.3%; Hui: 217, 87.9%). Two objective earthquake exposure experiences, including having lost a close family member or friend (Han: 283, 14.5%; Hui: 48, 19.4%) and having lost home or important belongings (Han: 1169, 59.9%; Hui: 170, 68.8%), yielded statistically significant differences between the ethnic Hui and Han child groups (Fig. 2A).

### Han-Hui differences in PTSD symptoms

The average UCLA PTSD-RI total score for the ethnic Hui child group (27.01 ± 9.24) was significantly higher than that of the ethnic Han group (25.12 ± 9.17) (t = −3.05, *p* = 0.002), as were those for avoidance/numbness score (Hui: 10.02 ± 4.82; Han: 9.04 ± 4.60, t = −3.12, *p* = 0.002) and arousal scores (Hui: 9.36 ± 3.64; Han: 8.79 ± 3.42, t = −2.44, *p* = 0.015) (Fig. 2B). There was a significant difference in the percentage of screening positive for D criteria (arousal symptoms) between the ethnic Hui (48.6%, 95% CI [42.3–54.9%]) and Han (41.9%, 95% CI [39.7–44.1%]) child groups (χ^2^ = 3.97, *p* = 0.046). The percentage of screening positive for B criteria (intrusion) (Hui: 45.7%, 95% CI [39.0–52.0%]; Han: 46.2%, 95% CI [44.0–48.0%], χ^2^ = 0.02, *p* = 0.89), C criteria (avoidance/numbness) (Hui: 19.8%, 95% CI [15.0–25.0%]; Han: 15.5%, 95% CI [14.0–17.0%], χ^2^ = 3.10, *p* = 0.08) and PTSD symptoms (Hui: 9.3%, 95% CI [5.7–13.0%]; Han: 8.0%, 95% CI [6.9–9.3%], χ^2^ = 0.99, *p* = 0.32) did not differ significantly between the Hui and Han ethnic child groups (Fig. 2C).

### Relationship between ethnic group and PTSD symptoms

We found a positive association between the ethnicity and the avoidance/numbness symptoms score (Tables 2 and 3). No association was observed between the ethnicity and the arousal symptoms score. The ethnic Hui minority was initially associated with a higher arousal symptoms score, higher PTSD total score, and higher percentage of screening positive for D criteria (arousal symptoms) following adjustment for demographic variables and personality traits (Models 2 and 3, Table 2). However, the significant association between the ethnicity and the percentage of screening positive for D criteria (arousal symptoms) (Model 1: OR = 1.31, 95% CI [1.00–1.71], *p* = 0.047) disappeared following further adjustment for objective earthquake exposure experiences (Model 4: OR = 1.32, 95% CI [1.00–1.75], *p* = 0.052). The significant associations between the ethnicity and the arousal symptoms score (Model 1: B = 0.57, 95% CI [0.11–1.02], *p* = 0.015) and PTSD total scores (Model 1: B = 1.89, 95% CI [0.67–3.10], *p* = 0.002) disappeared following further adjustment for subjective earthquake exposure experiences (Model 5: arousal symptoms, B = 0.41, 95% CI [−0.01 to 0.83], *p* = 0.056; PTSD, B = 1.00, 95% CI [−0.07 to 2.07], *p* = 0.066). Detailed information for Models 1 to 5 for significant PTSD symptoms is listed in the Supplemental Materials.

**Table 2:** Associations between the ethnicity and PTSD symptoms.

	Multiple linear regression									Multiple logistic regression	
	Avoidance/numbness symptoms score			Arousal symptoms score			PTSD total score			D criteria	
	B (95% CI)	*P*		B (95% CI)	*P*		B (95% CI)	*P*		OR (95% CI)	*P*
Model 1^a^											
Ethnic (the Hui minority)	0.97 [0.36–1.59]	0.002		0.57 [0.11–1.02]	0.015		1.89 [0.67–3.10]	0.002		1.31 [1.00–1.71]	0.047
Model 2^b^											
Ethnic (the Hui minority)	0.76 [0.16–1.37]	0.014		0.66 [0.21–1.12]	0.004		1.72 [0.51–2.94]	0.005		1.35 [1.03–1.76]	0.030
Model 3^c^											
Ethnic (the Hui minority)	0.83 [0.26–1.39]	0.004		0.63 [0.20–1.06]	0.004		1.71 [0.61–2.82]	0.002		1.36 [1.03–1.80]	0.029
Model 4^d^											
Ethnic (the Hui minority)	0.72 [0.16–1.29]	0.012		0.56 [0.14–0.98]	0.010		1.45 [0.37–2.54]	0.009		1.32 [1.00–1.75]	0.052
Model 5^e^											
Ethnic (the Hui minority)	0.61 [0.04–1.17]	0.035		0.41 [−0.01 to 0.83]	0.056		1.00 [−0.07 to 2.07]	0.066		1.21 [0.91–1.61]	0.186

**Note:**

a Model 1: only ethnic group is an independent variable.

b Model 2: adjusted for demographic variables (sex, age, residence, removal to another area after earthquake).

c Model 3, model 2 with additional adjustments for personality traits (subscale scores of JEPQ).

d Model 4, model 3 with additional adjustments for objective earthquake exposure experiences.

e Model 5, model 4 with additional adjustments for subjective earthquake exposure experiences.

Abbreviations: OR, odds ratio; 95% CI, 95% confidence interval.

**Table 3:** Associations between demographic variables, personality traits, earthquake exposure experiences and PTSD symptoms.

Independent variables	Multiple linear regression (Model 5)			Multiple logistic regression (Model 5)
	Avoidance/numbness symptoms score B (95% CI)	Arousal symptoms score B (95% CI)	PTSD total score B (95% CI)	D criteria OR (95% CI)
**Demographic variables**				
Sex (Male)	0.18 [−0.18 to 0.54]	−0.79 [−1.05 to −0.52]**	−1.23 [−1.91 to −0.55]**	0.70 [0.59–0.85]
Age (7–15 years)	−0.24 [−0.32 to −0.16]**	0.06 [0.00–0.12]*	−0.31 [−0.47 to −0.16]**	1.01 [0.97–1.05]
Ethnic (the Hui minority)	0.61 [0.04–1.17]*	0.41 [−0.01 to 0.83]	1.00 [−0.07 to 2.07]	1.21 [0.91–1.61]
Residence (Township)	−0.23 [−0.88 to 0.41]	−0.27 [−0.75 to 0.21]	−0.58 [−1.80 to 0.65]	0.91 [0.66–1.27]
Removal to another area after earthquake	0.03 [−0.33 to 0.40]	−0.16 [−0.43 to 0.12]	−0.04 [−0.73 to 0.65]	1.02 [0.85–1.23]
**Personality traits**				
*P* score	0.03 [0.02–0.05]**	−0.02 [−0.03 to −0.01]**	0.03 [−0.01 to 0.06]	0.99 [0.98–0.99]**
E score	−0.05 [−0.07 to −0.03]**	0.00 [−0.01 to 0.01]	−0.05 [−0.08 to −0.02]**	1.01 [1.01–1.02]**
*N* score	0.14 [0.12–0.16]**	0.12 [0.11–0.14]**	0.35 [0.31–0.39]**	1.05 [1.04–1.07]**
L score	0.03 [0.01–0.05]**	0.01 [0.00–0.02]	0.08 [0.04–0.11]**	0.98 [0.97–0.99]**
**Earthquake exposure experiences**				
**Objective**				
Lost home or important belongings	0.55 [0.17–0.92]**	0.59 [0.32–0.87]**	1.53 [0.82–2.23]**	1.37 [1.13–1.66]**
Close family member or friend was injured	0.63 [0.21–1.05]**	−0.10 [−0.41 to 0.21]	1.29 [0.50–2.08]**	1.06 [0.86–1.31]
Lost close family member or friend	0.64 [0.11–1.17]*	0.08 [−0.31 to 0.48]	1.13 [0.12–2.14]*	0.88 [0.67–1.16]
Saw any one dead or injured	−0.08 [−0.46 to 0.31]	0.45 [0.17–0.74]**	0.44 [−0.30 to 1.17]	1.19 [0.97–1.45]
Injured	0.33 [−0.42 to 1.07]	0.60 [0.04–1.15]*	1.50 [0.08–2.91]*	1.15 [0.78–1.67]
**Subjective**				
Trapped for a longer time	0.34 [−0.04 to 0.73]	0.47 [0.18–0.76]**	1.20 [0.47–1.93]**	1.40 [1.15–1.70]**
Felt extreme panic or fear	0.36 [−0.12 to 0.84]	0.71 [0.35–1.07]**	2.15 [1.24–3.07]**	1.40 [1.08–1.81]*
Felt unable to escape from the disaster	0.57 [0.19–0.94]**	0.52 [0.24–0.80]**	1.91 [1.20–2.62]**	1.36 [1.12–1.64]**
Felt one’s own or others’ life to have been in danger	0.09 [−0.31 to 0.49]	0.25 [−0.05 to 0.55]	0.23 [−0.53 to 1.00]	1.21 [0.98–1.49]
Felt panic of others	0.52 [−0.02 to 1.05]	0.31 [−0.09 to 0.71]	1.44 [0.42–2.45]**	1.39 [1.04–1.85]*
Constant	1.66 [−0.38 to 3.70]	0.55 [−0.97 to 2.07]	1.63 [−2.25 to 5.51]	0.04

**Note:**

* *p* < 0.05.

** *p* < 0.01.

Abbreviations: OR, odds ratio; 95% CI, 95% confidence interval; *P*, psychoticism/socialization subscale; E, extraversion/introversion subscale; *N*, neuroticism/stability subscale; L, lie subscale.

The associations between demographic variables, personality traits, earthquake exposure experiences and the PTSD symptoms are listed in Table 3. Sex was not related to the avoidance/numbness symptoms score, but was related to the arousal symptoms score. Age and the E subscale score were negatively associated with the avoidance/numbness symptoms score. The P, E, N and L subscale scores were positively associated with the avoidance/numbness symptoms score. Three objective earthquake exposure experiences (including lost home or important belongings, close family member or friend was injured, lost close family member or friend) and one subjective earthquake exposure experience (felt unable to escape from the disaster) were associated with the avoidance/numbness symptoms score.

## Discussion

This is the first study to investigate the differences of PTSD symptoms between the ethnic Hui minority and Han majority child earthquake survivors in China. We found that the ethnic Hui children showed more severe avoidance/numbness and arousal symptoms. The percentage of screening positive for D criteria (arousal symptoms) in the ethnic Hui children was higher than that of the ethnic Han children. The association between the ethnicity and avoidance/numbness symptoms persisted even with adjustment of demographic variables, personality traits, and earthquake exposure experiences.

When we investigated the association between the ethnicity and PTSD symptoms with the consideration of other confounders by regression analysis, we only observed an association between the severity of avoidance/numbness symptoms and ethnicity following adjustment of demographic variables, personality traits, and earthquake exposure experiences. One previous study found that ethnicity was associated with PTSD symptoms (Das-Munshi et al., 2016). However, another study did not identify an association between ethnicity and PTSD symptoms (Andrews et al., 2015). We think that there are two possible explanations for these different set of findings. First, the types of trauma were different. The subjects of our study experienced a disaster, while the subjects of the research conducted by *Andrews* and *Das-Munshi* did not (Andrews et al., 2015). Second, personality traits were adjusted in this study and not in previous studies. To our knowledge, this study is the first to investigate the differences in the PTSD symptoms between ethnic Hui and Han children. The possibility of disparate experiences based on ethnicity merits greater attention in future research.

We found that there was no association between the severity of arousal symptoms and ethnicity following the adjustments for subjective earthquake exposure experiences, in contrast to the association between the severity of avoidance/numbness symptoms and the ethnicity. Only one subjective earthquake exposure experience was correlated with avoidance/numbness symptoms, whereas three subjective earthquake exposure experiences were correlated with arousal symptoms. These findings suggest that the subjective earthquake exposure experiences were more closely related to arousal than avoidance/numbness symptoms. Moreover, the initial significant association between the PTSD total score and ethnicity also disappeared while adjusting for the subjective earthquake exposure experiences. This suggests that the subjective exposure experiences played an important role in the severity of PTSD symptoms, a finding consistent with previous studies (Chen et al., 2017; Trickey et al., 2012). These findings reveal that the amelioration of subjective earthquake exposure experiences might improve PTSD symptoms. Psychotherapy guidelines recommend trauma-focused cognitive behavioural therapy (TF-CBT) as the first-line treatment for children and adolescents suffering from PTSD (ISTSS, 2019; NICE, 2018). Trauma-focused psychotherapy directly addresses memories of the traumatic event or thoughts and feelings related to the traumatic event (Watkins, Sprang & Rothbaum, 2018). Therefore, the study’s findings of this study could provide useful information for TF-CBT interventions for the PTSD treatment of ethnic Hui children.

Ethnic Hui children exhibited significantly more than the ethnic Han children in three subjective earthquake exposures: having felt unable to escape from the disaster, having felt extreme panic or fear and having been trapped for a longer time. These three subjective exposures were related to the perceived uncontrollability of a stressor and were frequently reported as crucial risk factors of PTSD (Lubit et al., 2003; McDermott & Palmer, 2002), and proved even more important than objective exposures (Bomyea, Amir & Lang, 2012; Goldsmith et al., 2013; Trickey et al., 2012). Although the ethnic Hui and Han children have lived together in the same area, the ethnic Hui children share a belief of Islam (Han & Northoff, 2008); the ethnic Han children did not necessarily have religious beliefs. The ethnic Han children are shaped by Han culture, which values attention to relations and contexts than to salient objects (Han & Northoff, 2008) and thus might alleviate their negative perception of the earthquake. However, self-identification of being ethnic minorities may increase minority’s sense of uncontrollability against natural disasters (Das-Munshi et al., 2016; Han et al., 2013). Therefore, efforts of strengthen children’s integration with society and providing more social support could decrease their sense of uncontrollability.

We found females were more likely to present severe arousal symptoms than males. Age was negatively correlated with the severity of avoidance/numbness symptoms and arousal symptoms in child survivors. Females and younger age also have been reported as risk factors in many previous studies (Giannopoulou et al., 2006; Iwadare et al., 2014; Liu et al., 2010; Ma et al., 2011; Roussos et al., 2005). However, some scholars have argued against that association (Kolaitis et al., 2003; Pynoos et al., 1993; Yang et al., 2011). A meta-analysis showed that being female represented a small risk factor for PTSD and that younger age was unrelated to PTSD (Trickey et al., 2012).

Our study also found that the personality characteristics—including psychoticism, neuroticism and introversion traits—correlated positively with the severity of avoidance/numbness and arousal symptoms. These findings were consistent with previous research that indicated that psychoticism and neuroticism were risk factors for PTSD (Casella & Motta, 1990; Eysenck & Eysenck, 1985; Goldberg, 1993; Holeva & Tarrier, 2001; Lauterbach & Vrana, 2002; O’Toole et al., 1998). Our findings with regard to extraversion were consistent with the results from previous studies that served to identify a negative relationship with PTSD symptoms (Jaksic et al., 2012; Shakespeare-Finch, Gow & Smith, 2005). However, they differed from studies that showed no relationship between extraversion and PTSD (Lawrence & Fauerbach, 2003; Schnurr & Vielhauer, 1999). We also found that deception characteristics correlated with more severe avoidance/numbness symptoms. However, few studies have reported on the L subscale of the JEPQ (Eysenck, Barrett & Saklofske, 2020). Therefore, this result needs to be further examined in future studies.

This study had several limitations. First, to the fact that all of the participants were from the same area helped to match the macro-level environmental factors but led to unequal group sizes and a decrease in the sample size. Second, the interpretation of the results could be limited to a specific time period. This research consisted of a secondary analysis of a sub-sample from a large cross-sectional survey conducted in 2009, one year after the Wenchuan earthquake. Therefore, the differences identified between the two ethnic groups could have changed since 2009. Possible changes that occurred during the past twelve years could impact practical contributions of this study. We plan to conduct further research to determine if the differences identified between the two groups persisted over time.

## Conclusions

Our results showed that subjective earthquake exposure experiences mainly affected the association between ethnicity and PTSD symptoms between the ethnic Hui and Han children in China. The findings provide support for the recommendation that TF-CBT should be the first-line treatment for children and adolescents who suffer from PTSD. Therefore, the TF-CBT could also be an effective treatment for Chinese ethnic Hui and Han children who suffer from PTSD. Future research could be designed to examine whether cultural differences in perceptions and interpretations may account for variations in subjective experiences.

## Supplemental Information

10.7717/peerj.11967/supp-1 Supplemental Information 1 Detail information of models 1 to 5 for significant PTSD symptoms.

10.7717/peerj.11967/supp-2 Supplemental Information 2 Demographic, JEPQ, PsySTART Rapid Triage System items and UCLA PTSD-RI scores of 2198 children survivors after Wenchuan earthquake.

## Additional Information and Declarations

### Data Availability

The following information was supplied regarding data availability: Raw data is available as a Supplemental File.
